# A retrospective study on application of a classification criterion based on relative intervertebral tension in spinal fusion surgery for lumbar degenerative diseases

**DOI:** 10.1186/s12893-023-01968-x

**Published:** 2023-03-30

**Authors:** Yang Hou, Hongyang Shi, Tianyi Zhao, Haoyang Shi, Jiangang Shi, Guodong Shi

**Affiliations:** Department of Orthopaedic Surgery, Changzheng Hospital, Second Military Medical University, No. 415 Fengyang Rd, Shanghai, 200003 China

**Keywords:** Lumbar interbody fusion, Relative intervertebral tension, Fusion rate, Cage migration, Cage subsidence

## Abstract

**Background:**

As an important part of spinal fusion procedure, the selection of fusion cage size is closely related to the curative effect of the surgery. It mainly depends on the clinical experience of surgeons, and there is still a lack of objective standards. The purpose of this study is to propose the concept of relative intervertebral tension (RIT) for the first time and its grading standards to improve the surgical procedures of lumbar interbody fusion.

**Methods:**

This retrospective study was conducted from January 2018 to July 2019. A total of 83 eligible patients including 45 men and 38 women with lumbar degenerative disease underwent transforaminal lumbar interbody fusion (TLIF) were included in this study. A total of 151 fusion segments were divided into group A, group B and group C according to the grading standards of RIT. In addition, parameters of intervertebral space angle (ISA), intervertebral space height (ISH), intervertebral space foramen (IFH), fusion rates, cage-related complications and cage heights were also compared among the three groups.

**Results:**

The ISA in group A was the smallest among three groups in contrast with group C with largest ISA at the final follow-up(*P* < 0.05). The group A presented the smallest ISH and IFH values(*P* < 0.05), compared with group B with the largest ISH and IFH values(*P* < 0.05). These two parameters in the group C were in-between. The fusion rates of group A, group B and group C were 100%, 96.3% and 98.8% at the final follow-up, respectively. No statistical difference in fusion rates and cage-related complications occurred among the three groups(*P* > 0.05), and a certain correlation between ISH and RIT was also observed.

**Conclusions:**

The concept of RIT and the application of its clinical grading standards could simplify the surgical procedures of spinal fusion and reduce cage-related complications.

## Background

Lumbar interbody fusion(LIF)techniques have been widely used to treat degenerative lumbar diseases, which could effectively stabilize the pain motion segment, and indirectly reduce pressure, restore lordosis and correct deformity [1, 2]. LIF has been proved to have good clinical effect in the treatment of a variety of pathological spinal diseases, such as spinal stenosis, lumbar spondylolisthesis, recurrent nucleus pulposus protrusion, degenerative disc disease, spinal deformity, and trauma [3–5]. Posterior approaches can provide an excellent exposure of the nerve roots and direct decompression of the spinal cord. In addition, the posterior approach conforms to the operating habits of most spine surgeons and has a short learning curve, so it is widely used in clinical practice.

During the LIF surgery, most scholars adopt axial compression fixation for cage to increase the friction between cage and adjacent vertebral bodies to prevent its loosening or displacement at present [6, 7]. Some scholars also believe that cage can ensure relative stability without axial compression, because pedicle screws are enough to maintain the mechanical stability of lumbar spine in a short time. Cage is not easy to migrate or even fall out in the intervertebral space. With the extension of time, cage can fuse with the upper and lower vertebral bodies into a mechanical whole. In addition, non-compression of intervertebral space can maintain the height of intervertebral space and intervertebral foramen, while excessive axial compression of intervertebral space is bound to reduce the height of intervertebral space and intervertebral foramen, resulting in aggravation of nerve root compression [8]. Therefore, whether posterior axial compression is required after cage placement during lumbar interbody fusion surgery is still controversial.

We believe that the key to solve this problem lies in how to select the appropriate size of cage without posterior axial compression during lumbar interbody fusion surgery, so that it can maintain a certain degree of tension with adjacent vertebral bodies without displacement. At the same time, it can also avoid excessive expansion of intervertebral space caused by too large size of cage, resulting in postoperative neurological symptoms. In addition, the selection of a cage with appropriate size plays a very important role in improving lumbar lordosis, preventing postoperative cage subsidence or displacement, and reducing nerve root damage [9, 10].

However, there is a lack of quantifiable criteria for the selection of the cage size during interbody fusion in previous literature reports. Basically, the selection of the proper cage is based on the preoperative imaging examination of the patient and the personal clinical experience of the spine surgeon. Therefore, it is difficult to effectively reduce the complications related to the fusion cage after the operation. Based on the long-term experience in surgical treatment of degenerative lumbar diseases, we firstly proposed the concept of relative intervertebral tension (RIT) to guide the selection of fusion cage during the interbody fusion. We believe that the selection criteria of fusion cage based on RIT could reduce the cage-related complications and improve the surgical effects.

## Methods

This is a retrospective study that has been approved by the institutional review board of our hospital. The informed consent was obtained from each eligible patient preoperatively. This study included the eligible patients with multilevel lumbar degenerative diseases who received the TLIF in our department from January 2018 to July 2019. The patients’ inclusion criteria include the following: (1) symptoms of neurogenic claudication (pain, tingling, or cramping in the lower back and one or both legs, hips, and buttocks. Weakness or heaviness in the legs may also occur); (2) No improvement in symptoms after 3 months of conservative treatment; (3) No more than four operative levels with imaging examination; (4) all cases were followed up for more than 1 year.

The exclusion criteria include the following: (1) history of spinal fractures or surgery; (2) congenital spinal deformity, tumor, tuberculosis, or metabolic bone disease; (3) combined with neurological disease such as Parkinson’s, Alzheimer’s dementia, etc.; (4) severe diabetes and other metabolic disease without regular treatment; (5) a history of psychosis; (6) a history of alcoholism or drug addiction; 7) any serious general illness (e.g., heart failure, HIV).

### Surgical technique

The surgical procedures of TLIF have been well described by previous literatures [11, 12]. The patient was placed in prone position after general anesthesia. A posterior midline incision is made over the lumbar spine and the paraspinal muscles are detached from the spinous process, lamina, facet capsules and the transverse processes. A uni- or bilateral inferior facetectomy is performed followed by superior facet resection, discectomy, interbody cage (Shandong cansun Medical Devices Co, China or Double Medical Technology Inc, China) implantation, and pedicle screw placement. After the operation, hemostasis, irrigation, placement of negative pressure drainage tube and suture were performed. The patients were treated with anti-infection, nutrition, and rehabilitation training. All surgeries were performed by the corresponding author. Following surgery, all patients were immobilized in a waist harness for 8–12 weeks.

### Relative intervertebral tension (RIT)

**Fig. 1:**
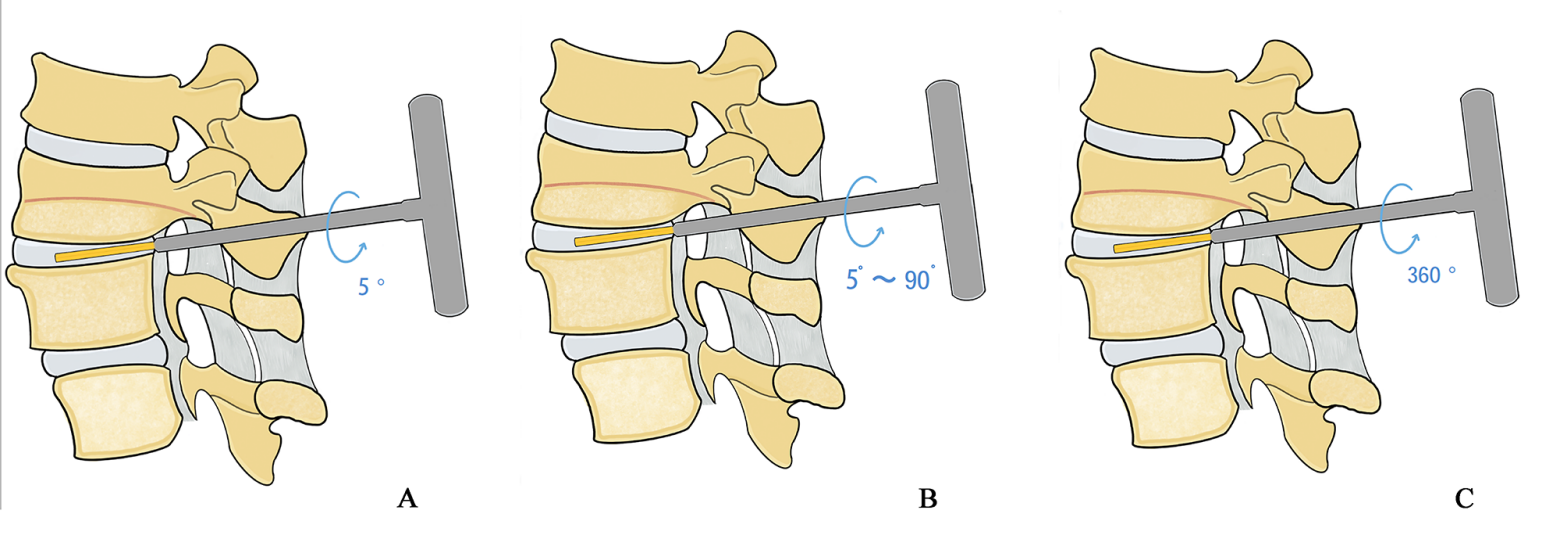
Illustrations of grade I (**A**), grade II (**B**) and grade III (**C**) of RIT, respectively

**Fig. 2:**
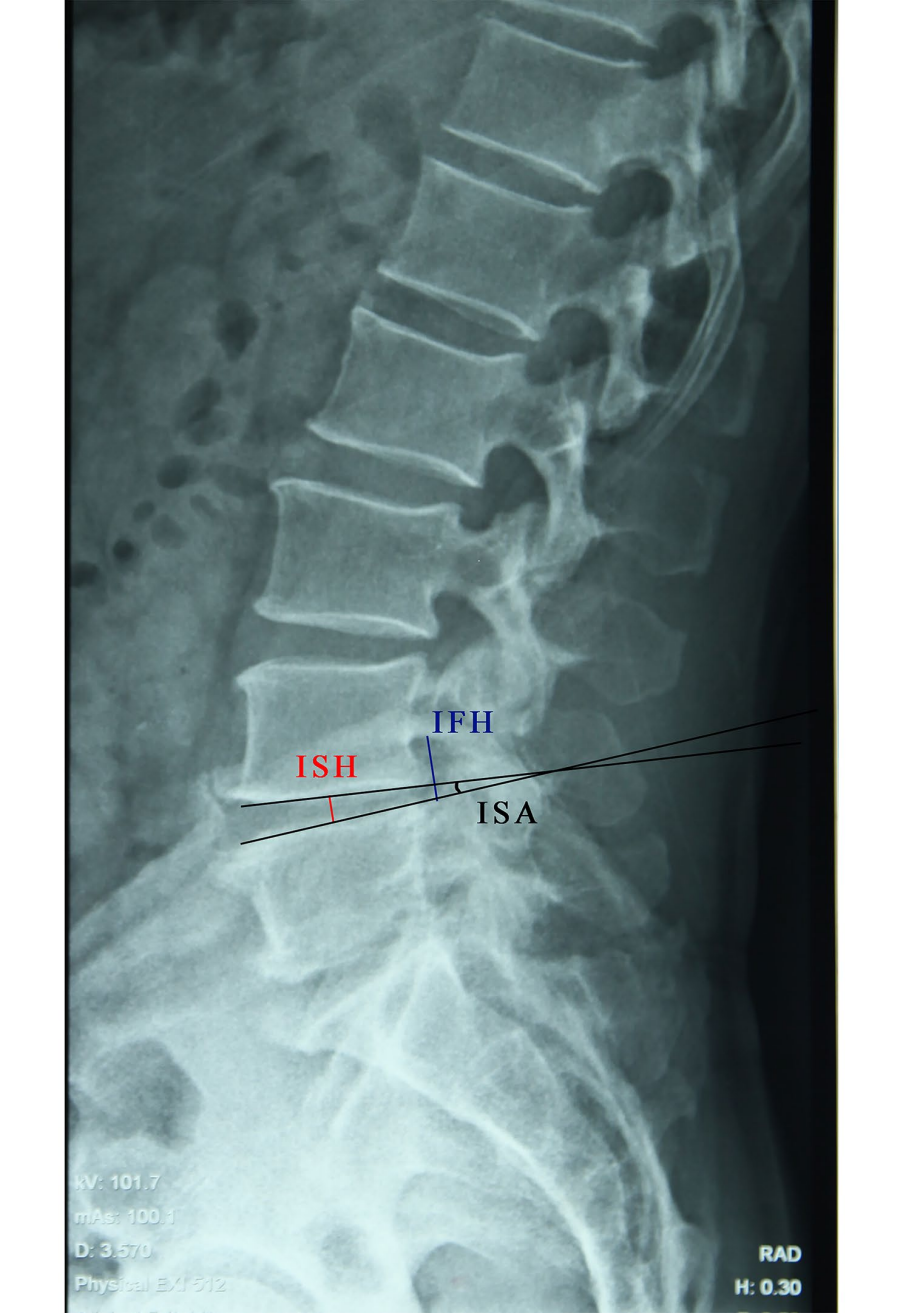
Diagram of the measurement of ISH (red line), ISA (black line), and IFH (blue line)

**Fig. 3:**
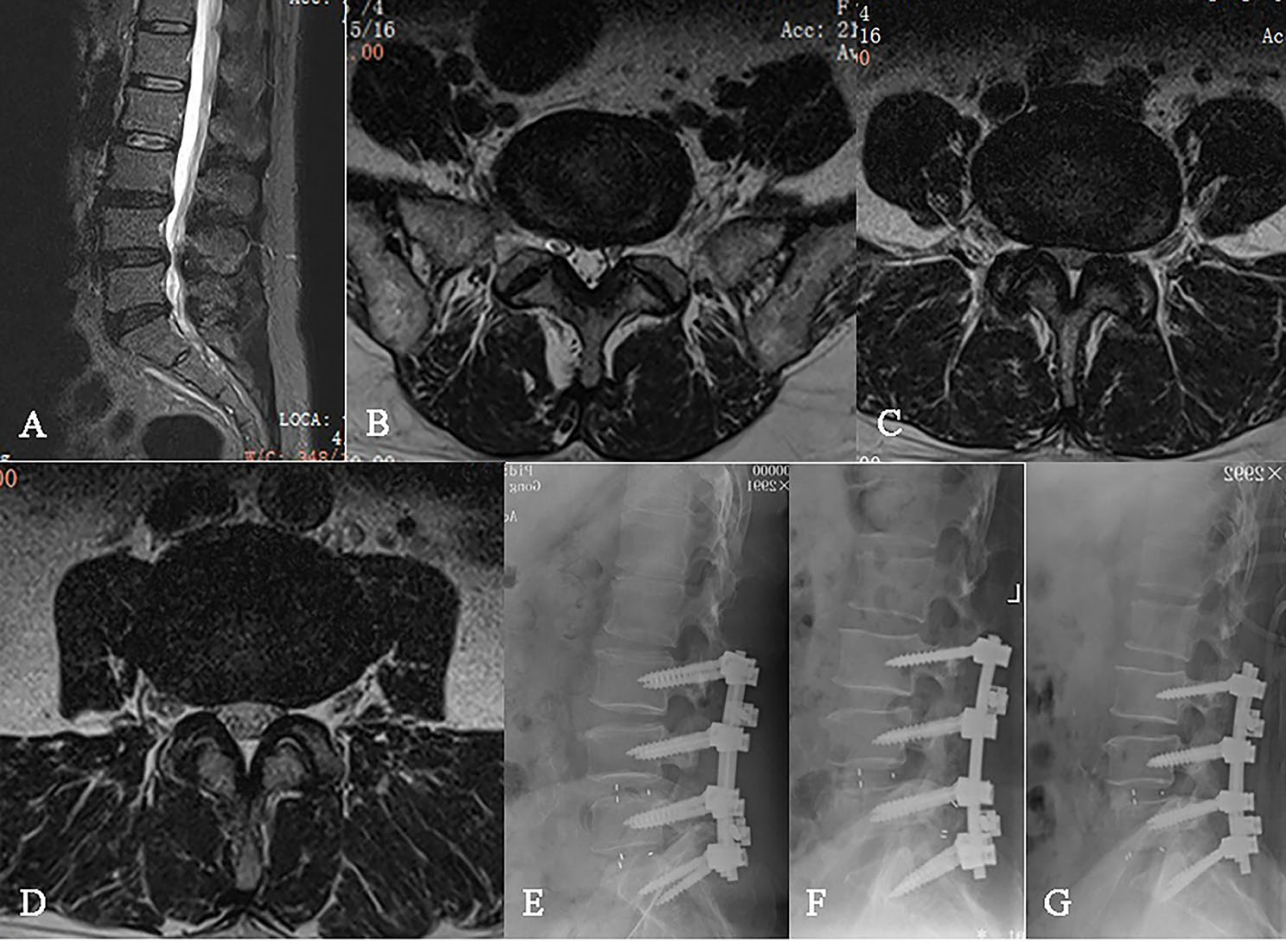
Preoperative lateral MRI of a 62-year-old female with lumbar spinal stenosis (**A**); MRI of the patient at L5/S1 (**B**), L4/5 (**C**), and L3/4 (**D**) levels; Radiographic view of the patient at 3rd day (**E**), 1st month (**F**) after first operation and after second operation (**G**)

We firstly proposed the concept of RIT which was defined as the resistance encountered when the distractor rotates in the intervertebral space during the fusion operation. The strength of RIT depends on two factors: the intervertebral space height (ISH) and the cage height. When the ISH remains constant, the higher the cage height, the greater the strength of RIT; When the cage height is constant, the lower the ISH, the greater the strength of RIT. According to the changes of RIT strength, we propose the grading standards of RIT. (1) Grade I: the distractor rotates no more than 5º in the intervertebral space and the RIT can resist the pulling force of the distractor (Fig. 1A); (2) Grade II: The rotation range of the distractor in the intervertebral space is more than 5º and less than 90º. The RIT can resist the pulling force of the distractor (Fig. 1B); (3) Grade III: The distractor can be rotated 360 º in the intervertebral space and the RIT cannot resist the pulling force of the distractor (Fig. 1C).

During the interbody fusion, the distractors from the smallest size(width = 7 mm) are inserted one after the other on alternate sides of the disc. If the RIT belongs to grade I, the intervertebral space should be moderately distracted, and then the cage with the corresponding size should be inserted without the intervertebral compression performance. If the RIT belongs to grade II, the cage with the corresponding size could be inserted without the intervertebral compression performance. If the RIT belongs to grade III, the corresponding size of cage could be inserted, and the intervertebral compression should be performed at the same time. Alternatively, a larger size of cage could be selected until the RIT strength meets the grade I or grade II criteria. According to the RIT grading standards, the fusion segments in this study were divided into group A (Grade I), group B (Grade II) and group C (Grade III), respectively.

### Clinical evaluation

The Visual analogue scale (VAS) score, Oswestry disability index (ODI), blood loss, and operative time of patients were recorded at each follow-up. The fusion rates and prosthesis related complications including prosthesis migration or subsidence at each fusion level were also recorded. The follow-up examinations were performed on day 1, and then at the 3- and 6-month follow-ups. The subsequent follow-up examinations were performed at 6-month intervals. At each follow-up visit, patients were required to undergo the imaging examinations and complete an assessment questionnaire.

### Imaging analysis

(1) Intervertebral space angle (ISA): the angle between the upper and lower endplate of the intervertebral space. (2) Intervertebral space height (ISH): the mean of anterior and posterior ISH. (3) Intervertebral foramen height (IFH): the distance between the lower margin of the superior pedicle and vertebral body connection and the upper margin of the inferior pedicle and vertebral body connection (Fig. 2) (4); Bone graft fusion was evaluated according to the criterion reported by Bridwell et al [13]. To correct the intra-observer and inter-observer reliability of the radiological measurements, three experienced observers were assigned to independently evaluate the radiographs of the patients. Each of them took measurements three times, and the mean values were used for statistical analysis.

### Cage-related complications

The cage migration and subsidence were evaluated in each fusion level according to the radiological examinations at each follow-up time point. Cage migration was defined as posterior movement of the cage past the posterior wall of the vertebral body. Correct initial positioning of the cages immediately postoperatively was confirmed by plain X-ray, whereas postoperative cage migration was determined by CT scan as well as plain films [14]. Cage subsidence was evaluated using lateral radiographs and was defined as more than 2-mm migration of the cage into the adjacent vertebral body [15, 16].

### Statistical analysis

An independent sample t test or Wilcoxon rank-sum test was used to investigate whether statistical differences of the results existed among the groups postoperatively, and multiple comparisons between groups were performed using the Student-Newman-Keuls (SNK) test. The chi-square test was used in the comparisons of the complication rates between groups. All the statistical tests were completed by the Statistical Package for Social Sciences software for Windows (Ver. 26.0; SPSS Inc, Chicago, IL), and the difference was considered to be statistically significant at the *P* < 0.05 level.

## Results

### Patient demographics and surgical outcome

A total of 113 cases were reviewed during the study period based on data of patients from our department. Among them, 22 cases were excluded because they failed to meet the inclusion criteria and 8 cases were excluded because of loss to follow-up. Finally, there were 83 patients (mean age, 61.5 ± 9.4y; 45 men, 38 women) included and the follow-up period was 19.1 ± 3.8 months. The eligible cases in this study included 33 cases with lumbar disc herniation, 41 cases with lumbar spinal stenosis, and 9 cases with lumbar spondylolisthesis. Among them, there were 15 cases with one operated level, 42 cases with two operated levels, 18 cases with three operated levels and 8 cases with four operated levels. There was a total of 151 interbody fusion segments which included 11 segments in group A, 54 segments in group B, 86 segments in group C according to the grading standards of RIT. The average surgery time and blood loss of all cases were 135.5 ± 12.1 min and 310 ± 41.2 ml, respectively. The VAS scores significantly decreased from 7.91 ± 0.64 to 1.39 ± 0.51 at the final follow-up (*P* < 0.05) and the ODI scores significantly decreased from 54.29 ± 6.18 to 12.05 ± 1.86 at the final follow-up (*P* < 0.05, Table 1), respectively.

**Table 1:** Preoperative clinical features of the eligible cases in this study

Age	61.5 ± 9.4
Sex(n)	45 males, 38 females
Follow-up period(month)	19.1 ± 3.8
One operated level	15
Two operated levels (two fusion levels)	42
Three operated levels (two fusion levels)	18
Four operated levels (two fusion levels)	8
Lumbar disc herniation	33
Lumbar spinal stenosis	41
Lumbar spondylolisthesis	9
Operation time(min)	135.5 ± 12.1
Blood loss(ml)	310 ± 41.2
Cage height (8 mm)	14
Cage height (9 mm)	32
Cage height (10 mm)	41
Cage height (11 mm)	6
Cage height (12 mm)	58
Grade I	11
Grade II	54
Grade III	86
Preoperative VAS	7.91 ± 0.64
Postoperative VAS at final follow-up	1.39 ± 0.51
Preoperative ODI	54.29 ± 6.18
Postoperative ODI at final follow-up	12.05 ± 1.86

### Radiographic results

The ISA in the group A significantly improved from 6.19 ± 4.01° to 7.94 ± 3.55° at the final follow-up (*P* < 0.05, Table 2). In the meanwhile, the group B showed a significant increase of ISA from 6.24 ± 3.55° to 9.08 ± 3.44° at the final follow-up (*P* < 0.05). The ISA in the group C significantly increased from 6.35 ± 3.78° to 10.29 ± 3.41°(*P* < 0.05). The ISH of group A, group B and group C increased from 4.52 ± 1.21 mm to 9.92 ± 0.85 mm(*P* < 0.05), 8.97 ± 0.82 mm to 12.74 ± 0.88 mm(*P* < 0.05), and 9.02 ± 0.69 mm to 10.93 ± 0.91 mm(*P* < 0.05) at the final follow-up, respectively. The IFH showed a significant increase from 9.67 ± 1.07 mm to 15.19 ± 1.06 mm(*P* < 0.05), 12.44 ± 1.52 mm to 18.09 ± 1.13 mm(*P* < 0.05), 12.51 ± 1.23 mm to 16.41 ± 1.01 mm(*P* < 0.05) at the final follow-up, respectively. Significant differences of the results occurred among the three groups (*P* < 0.05), and the multiple comparisons among the three groups also revealed statistical difference (*P* < 0.05). The ISA in group A was the smallest among three groups in contrast with group C with largest ISA at the final follow-up (*P* < 0.05). The group A presented the smallest ISH and IFH values, compared with group B with the largest ISH and IFH values (*P* < 0.05). These two parameters in the group C were in-between. The fusion rates of the group A, B and C were 100%, 96.3% and 98.8% at the final follow-up, respectively (Table 3). Multiple comparisons using chi-square test indicated that there was no statistical difference in fusion rates between groups(*P* > 0.05).

**Table 2:** Radiographic results at different follow-up time in fusion levels with different types of IST

	Group A(n = 11)	Group B(n = 54)	Group C(n = 86)
Preoperative ISA (º)	6.19 ± 4.01	6.24 ± 3.55	6.35 ± 3.78
Postoperative ISA at 3th month follow-up(º)	8.12 ± 3.97	9.33 ± 4.01	10.72 ± 3.74
Postoperative ISA at 6th month follow-up(º)	8.09 ± 3.77	9.27 ± 3.89	10.59 ± 3.52
Postoperative ISA at 12th month follow-up(º)	8.01 ± 3.89	9.19 ± 3.58	10.37 ± 3.46
Postoperative ISA at final follow-up(º)	7.94 ± 3.55	9.08 ± 3.44	10.29 ± 3.41
P value within group	*P* < 0.05	*P* < 0.05	*P* < 0.05
Preoperative ISH (mm)	4.52 ± 1.21	8.97 ± 0.82	9.02 ± 0.69
Postoperative ISH at 3th month follow-up(mm)	10.14 ± 0.93	13.43 ± 0.84	11.39 ± 0.86
Postoperative ISH at 6th month follow-up(mm)	10.09 ± 0.87	13.02 ± 0.81	11.21 ± 0.74
Postoperative ISH at 12th month follow-up(mm)	10.01 ± 0.75	12.91 ± 0.90	11.15 ± 0.63
Postoperative ISH at final follow-up(mm)	9.92 ± 0.85	12.74 ± 0.88	10.93 ± 0.91
P value within group	*P* < 0.05	*P* < 0.05	*P* < 0.05
Preoperative IFH (mm)	9.67 ± 1.07	12.44 ± 1.52	12.51 ± 1.23
Postoperative IFH at 3th month follow-up(mm)	15.42 ± 1.04	18.33 ± 1.32	16.79 ± 1.18
Postoperative IFH at 6th month follow-up(mm)	15.39 ± 1.08	18.27 ± 1.24	16.55 ± 1.04
Postoperative IFH at 12th month follow-up(mm)	15.28 ± 1.22	18.16 ± 1.18	16.49 ± 1.07
Postoperative IFH at final follow-up(mm)	15.19 ± 1.06	18.09 ± 1.13	16.41 ± 1.01
P value within group	*P* < 0.05	*P* < 0.05	*P* < 0.05

**Table 3:** Results of fusion and cage-related complications in fusion segments Please pay attention to the letter (a) marked on each number. If there are the same letter, it means that the comparison between the two groups is not statistically significant (*P* > 0.05)

	Group A(n = 11)	Group B(n = 54)	Group C(n = 86)
Fusion rates	11(100%) ^a^	52(96.30%) ^a^	85(98.84%) ^a^
Migration of prosthesis(n)	0 ^a^	1(1.85%) ^a^	1(1.16%) ^a^
Subsidence of prosthesis(n)	3(27.27%) ^a^	11(20.37%) ^a^	19(22.09%) ^a^

### Cage-related complications

**Fig. 4:**
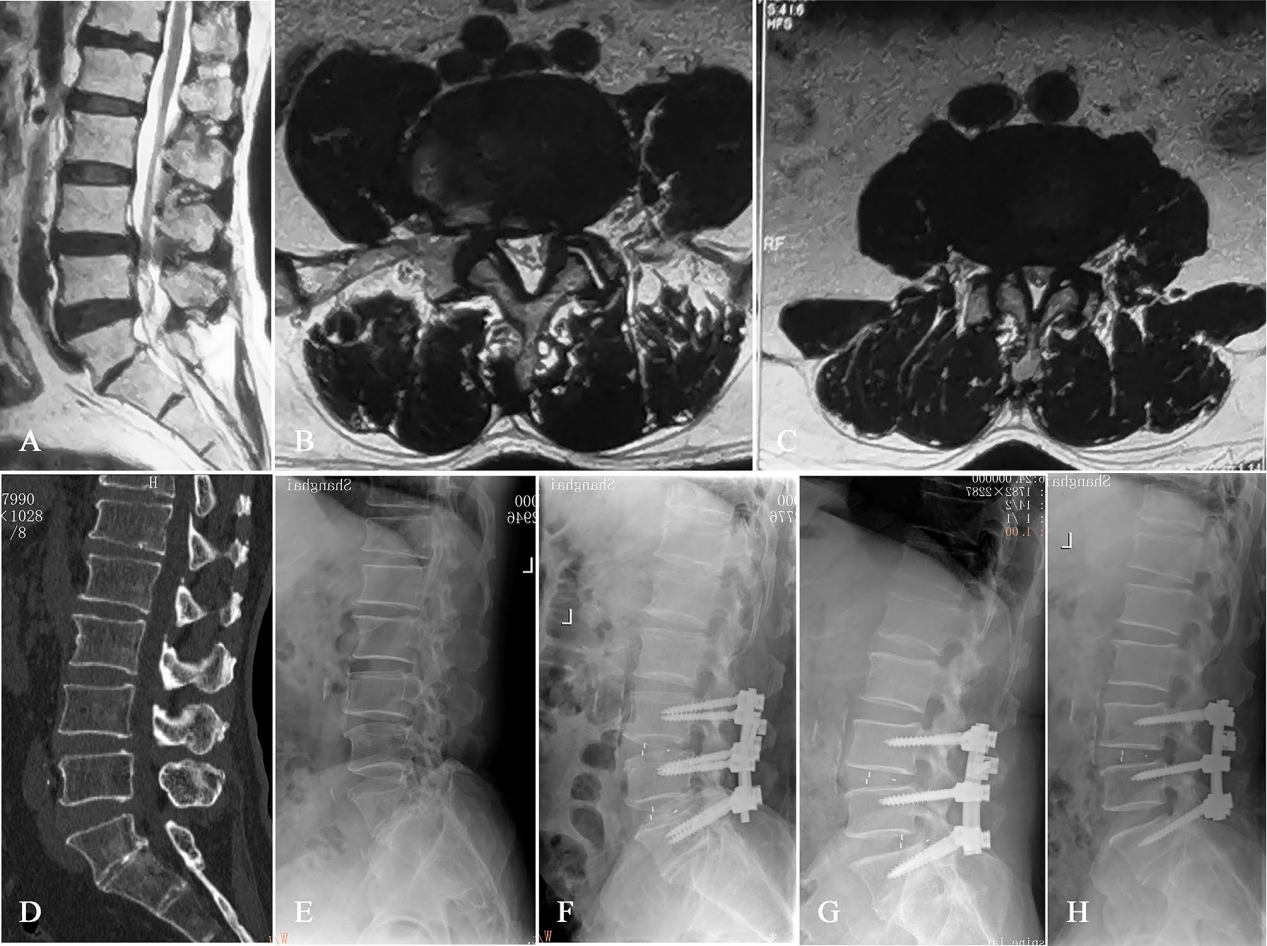
Imaging examinations (**A-E**) of a 66-year-old male showed lumbar spinal stenosis at L4/5 (**B**) and L3/4 (**C**) levels; Radiographic view of the patient at 3rd day (**F**), 3rd month (**G**) after first operation and after second operation (**H**)

There was no case with prosthesis migration in group A. One case with prosthesis migration occurred in group B (1.85%) and group C (1.16%), respectively. Both patients underwent secondary surgery. For one patient, a cage with larger size was selected for re-implantation (Fig. 3). For the other patient, the displaced cage was removed, and autogenous bone grafts mixed with allografts were implanted into the intervertebral disc space (Fig. 4). Both patients finally achieved satisfactory clinical efficacy and recovery at the final follow-up. Prosthetic subsidence occurred in 3 cases, 11 cases and 19 cases in group A (27.27%), group B (20.37%) and group C (22.09%), respectively. The patients with prosthetic subsidence did not have any clinical symptoms, therefore no medical treatment was adopted. Multiple comparisons between groups using chi-square test showed no significant difference (*P* > 0.05).

### Relationship between cage height and RIT

The application of cages with different heights in each group is shown in Table 4. The group A included 11 cages (100%) with the height of 8 mm, which has statistically significant differences compared with the other two groups(*P* < 0.05). For cages with the height of 9 mm, 10 and 12 mm, statistically significant difference occurred between the group B and group C(*P* < 0.05). There are also statistically significant differences between group A and group C in cages with the height of 10 mm(*P* < 0.05), and between group A and group B in cages with the height of 12 mm(*P* < 0.05). No statistical difference occurred in other multiple comparisons (*P* > 0.05).

**Table 4:** Application of fusion cages with different heights in each group

	Group A(n = 11)	Group B(n = 54)	Group C(n = 86)
Cage height (8 mm, n = 14)	11(100%) ^a^	3(5.56%) ^b^	0^b^
Cage height (9 mm, n = 32)	0^a, b^	4(7.41%) ^b^	28(32.56%) ^a^
Cage height (10 mm, n = 41)	0^a^	8(14.81%) ^a^	33(38.37%) ^b^
Cage height (11 mm, n = 6)	0^a^	2(3.70%) ^a^	4(4.65%) ^a^
Cage height (12 mm, n = 58)	0^a^	37(68.52%)^b^	21(24.42%)^a^

Please pay attention to the letters (a, b) marked on each number. If there are the same letters, it means that the comparison between the two groups is not statistically significant (*P* > 0.05)

## Discussion

In the posterior lumbar interbody fusion, cage is usually inserted through the intervertebral space to increase the support capacity and mechanical stability of the lumbar anterior and middle column, increase the bone graft fusion area, and improve the fusion rate [17, 18]. At present, most scholars adopt axial compression fixation for cage to increase the friction between cage and adjacent vertebral bodies to prevent it from loosening or displacement [19].

It is very important for the selection of cage size during lumbar interbody fusion operation. If the cage size is too small, it may increase the risk of postoperative prosthesis displacement or subsidence [20, 21]. Even if axial compression is performed, it may cause relative stenosis of intervertebral space or intervertebral foramen, which could result in secondary compression of nerve roots leading to neurological symptoms of lower limbs [22]. If the cage size is too large, it could increase the difficulty of prosthesis placement, cause dural tear and nerve root injury, and may also damage endplates of adjacent vertebral bodies. In addition, previous study also indicated that cage of large size will increase the incidence of adjacent intervertebral disc degeneration [23]. At present, the selection of appropriate size of cage mainly depends on preoperative imaging examination and the experience of surgeons. Due to the lack of unified standard, it subjects to a certain degree of subjectivity for the selection of cage size during the operation, which may increase the complications of surgery.

The results of this study showed there was no significant difference in fusion rates among the three groups(*P* > 0.05). In the meanwhile, the ISA in group A was the smallest at the last follow-up(*P* < 0.05), while the ISA in group C was the largest at the last follow-up(*P* < 0.05). Due to the fact that posterior axial compression was only done in group C, this indicates that the posterior axial compression is beneficial to increase the ISA of the fusion segment. In addition, the restoration of the ISH is also beneficial to increase the ISA of the fusion segment. The ISH and IFH of group B at the last follow-up were significantly higher than those of the other two groups (*P* < 0.05), which showed that the fusion segments with grade II of RIT was helpful to maintain the ideal ISH and IFH, while fusion segments with RIT of grade III and posterior axial compression would reduce ISH and IFH.

In this study, the case with postoperative cage displacement in group C occurred in 1 month after operation, which was related to the improper selection of cage size and insufficient posterior axial compression. The case of cage displacement in group B occurred in 3 months after operation, which was related to the influence of insufficient treatment of cartilage endplate on bone fusion during operation. Therefore, we suggest that adequate posterior compression must be performed to prevent cage displacement for fusion segments with grade III of RIT. For fusion segments with grade II or grade I of RIT, posterior axial compression is unnecessary, and there is no significant increase in the incidence of postoperative cage displacement.

Cage subsidence is one of the common complications after lumbar interbody fusion, with a reported incidence of between 14.3% and 76.7% [15, 16, 24, 25]. Yao YC et al. [16] have demonstrated that the BMD, disc height, and cage position were the most significant risk factors that were negatively correlated with depth of cage subsidence. The current study showed that the cases with grade I of RIT presented significantly lower preoperative ISH than that of the other two groups, and highest incidence of postoperative cage subsidence among three groups. The results were consistent with those reported by Yao YC et al [16]. The narrow ISH in group A increases the difficulty of cage placement. If the direction of intervertebral space cannot be clearly identified during the surgery, the cage may be placed into the adjacent vertebral body, which means that the cage subsidence could occur intraoperatively. In addition, for the serious narrow intervertebral space, we usually adopt the distractor to properly expand the intervertebral space so that the cage can be implanted smoothly. In this process, it may increase the incidence of bony end plate injury, and ultimately lead to the occurrence of cage subsidence. We believe that the above factors are responsible for greater cage subsidence in group A. However, multiple comparisons between groups using chi-square test showed no significant difference, which could be related to the relatively small number of cases in group A that may influence the statistical power.

The cause that cages with the height of 8 mm mainly concentrated in group A is owing to the fact that the fusion segment with grade I of RIT is usually characterized by severe loss of intervertebral height. However, if the cage height is close to the average ISH of normal people, the grade of RIT does not increase with the increase of cage height. This demonstrates that there is a correlation between the RIT and ISH, however, other factors may also affect the strength of RIT. We believe that the RIT is also related to the flexibility of paravertebral muscles and ligaments, the spinal joint hyperplasia, and the formation of bone bridge. Therefore, we believe that the size of the cage should not be determined only by referring to the ISH on the preoperative X-ray, but also in combination with the actual situation during the operation.

## Conclusion

The RIT classification can provide a useful tool to help spine surgeons to select fusion cages of appropriate size intraoperatively, rather than relying on preoperative imaging data, which is more in line with clinical practical needs, making the selection of fusion cages easier and more standardized.

## Data Availability

The data that support the findings of this study are available on request from the corresponding author. The data are not publicly available due to privacy or ethical restrictions.
